# Time-Lagged t-Distributed Stochastic Neighbor Embedding (t-SNE) of Molecular Simulation Trajectories

**DOI:** 10.3389/fmolb.2020.00132

**Published:** 2020-06-30

**Authors:** Vojtěch Spiwok, Pavel Kříž

**Affiliations:** ^1^Department of Biochemistry and Microbiology, University of Chemistry and Technology, Prague, Czechia; ^2^Department of Mathematics, University of Chemistry and Technology, Prague, Czechia

**Keywords:** molecular dynamics, dimensionality reduction, trajectory analysis, tSNE, Time-lagged Independent Component Analysis

## Abstract

Molecular simulation trajectories represent high-dimensional data. Such data can be visualized by methods of dimensionality reduction. Non-linear dimensionality reduction methods are likely to be more efficient than linear ones due to the fact that motions of atoms are non-linear. Here we test a popular non-linear t-distributed Stochastic Neighbor Embedding (t-SNE) method on analysis of trajectories of 200 ns alanine dipeptide dynamics and 208 μs Trp-cage folding and unfolding. Furthermore, we introduce a time-lagged variant of t-SNE in order to focus on rarely occurring transitions in the molecular system. This time-lagged t-SNE efficiently separates states according to distance in time. Using this method it is possible to visualize key states of studied systems (e.g., unfolded and folded protein) as well as possible kinetic traps using a two-dimensional plot. Time-lagged t-SNE is a visualization method and other applications, such as clustering and free energy modeling, must be done with caution.

## 1. Introduction

The main goal of molecular simulations is identification of key states of studied systems and building thermodynamic and kinetic models of transitions between these states. Identification of key states is often based on some numerical descriptors known as collective variables. Distance between two atoms can be seen as one of the simplest collective variables. It can be used, for example, to distinguish between the bound and unbound state in a simulation of protein-ligand interaction. For some more complex processes it is necessary to use more complex collective variables.

Collective variables are in fact dimensionality reduction methods because they represent high dimensional structure of a molecular system using few numerical descriptors. It is therefore no surprise that general linear and non-linear dimensionality reduction methods have been applied on molecular simulation trajectories. Namely, principal component analysis (Amadei et al., 1993; Spiwok et al., 2007; Sutto et al., 2010) and its dihedral version (Mu et al., 2005), diffusion maps (Ferguson et al., 2010, 2011), sketch map (Ceriotti et al., 2011; Tribello et al., 2012), Isomap (Das et al., 2006; Brown et al., 2008; Spiwok and Králová, 2011), autoencoders (Chen and Ferguson, 2018), t-SNE (van der Maaten and Hinton, 2008; Duan et al., 2013; Tribello and Gasparotto, 2019) and others (Plaku et al., 2007; Stamati et al., 2010; Noé and Clementi, 2015) have been tested in analysis of trajectories, data compression or sampling enhancement.

Advantage of non-linear dimensionality reduction methods is their ability to describe more variance in data compared to linear methods with the same number of dimensions. This is especially true for t-distributed Stochastic Neighbor Embedding (t-SNE) (van der Maaten and Hinton, 2008). This method became highly popular in many fields, including data science, bioinformatics, and computational linguistics.

There are two features of t-SNE that contributed to its success. First, t-SNE converts high-dimensional points into low-dimensional points in a way to reproduce their proximity rather than distance. For example, for a bioinformatician analyzing genomic data to develop genomics-based diagnosis it is important that samples with the same diagnosis are close to each other after dimensionality reduction. It is unimportant how distant are samples with different diagnosis, provided that they are distant enough. In t-SNE the distances in the high-dimensional space *D*_*ij*_ = ||*X*_*i*_−*X*_*j*_|| are converted into proximities *p*_*ij*_ as:

(1) $$\begin{array}{r} p_{ij}=\frac{\exp\left(-D_{ij}^{2}/2\sigma_{i}^{2}\right)}{\sum_{k\neq i}\exp\left(-D_{ik}^{2}/2\sigma_{i}^{2}\right)}, \end{array}$$

where $\sigma_{i}^{2}$ is the variance of a Gaussian centered on a datapoint *X*_*i*_ (discussed later). The matrix of proximities is then symmetrized. Next, proximities in the low-dimensional space *q*_*ij*_ are calculated from distances in the low-dimensional space *d*_*ij*_ as:

(2) $$\begin{array}{r} q_{ij}=\frac{{\left(1+d_{ij}^{2}\right)}^{-1}}{\sum_{k\neq i}{\left(1+d_{ik}^{2}\right)}^{-1}}. \end{array}$$

Finally, positions of points in the low-dimensional space are optimized to minimize Kullback-Leibler divergences of *p*_*ij*_ and *q*_*ij*_ (a sort of a distance between proximities *p* and *q*).

The second advantage of t-SNE lies in the fact that it unifies density of low-dimensional points in the output space. This feature, which can be controlled by a parameter called perplexity, makes visual representation of points more effective. Perplexity is related to variances $\sigma_{i}^{2}$ of Gaussians centered on datapoints *X*_*i*_. Unification of densities is done by different variances $\sigma_{i}^{2}$. The user can specify the value of perplexity. t-SNE searches for optimal values of $\sigma_{i}^{2}$ in order to produce values of $2^{-\underset{j}{\sum }p_{ji}\log_{2}p_{ji}}$ to match the predefined perplexity. Low perplexity (e.g., 5) forces focus on local structure of the input data whereas larger perplexity (e.g., 50) takes more global structure into the account. As discussed later, this feature improves visualization by t-SNE but at the same time it complicates application in situations when preservation of densities is required.

Disadvantage of application of general dimensionality reduction methods on molecular simulation trajectories is that these methods pick the most intensive (in terms of changes of atomic coordinates) motions in the system. However, such motions are often not interesting. For example, such intensive motions may represent motions of disordered loops or terminal chains in proteins.

Instead, for building of thermodynamic and kinetic models or to enhance sampling it is useful to extract motions that occur most rarely, i.e., those with the highest barriers. This can be done by Time-lagged Independent Component Analysis (TICA) (Molgedey and Schuster, 1994; Perez-Hernandez et al., 2013; Schwantes and Pande, 2013). TICA extracts the most rarely occurring transitions in the molecular system because it correlates the state of the system with the state of the same system after a short delay (lag). This lag can be controlled.

Here we attempt to join the advantages of t-SNE and TICA into a single method of time-lagged t-SNE. The method was tested on two molecular trajectories—on 200 ns simulation of alanine dipeptide and 208.8 μs simulation of Trp-cage mini-protein folding and unfolding (trajectory kindly provided by DE Shaw Research) (Lindorff-Larsen et al., 2011).

## 2. Methods

Time-lagged t-SNE is inspired by implementation of TICA using the AMUSE algorithm (Hyvarinen et al., 2001). We start with atomic coordinates **X**(*t*) recorded over time *t*. First, coordinates are superimposed to reference coordinates of the system to eliminate translational and rotational motions. After that, time-averaged coordinates are subtracted, leading to atomic displacements **X′**(*t*). Next, its covariance matrix is calculated as:

(3) $$\begin{array}{r} C_{ij}^{{\text{X}}^{\prime }}=\langle X_{i}^{\prime }\left(t\right)X_{j}^{\prime }\left(t\right)\rangle , \end{array}$$

where *i* and *j* are indexes of atomic coordinates and 〈〉 denotes time-averaging. Next, covariance matrix is decomposed to a diagonal matrix with eigenvalues **λ**^**X′**^ (the square matrix with eigenvalues on diagonal and zeros elsewhere) and eigenvectors **W**^**X′**^ (the matrix with eigenvectors as columns):

(4) $$\begin{array}{r} {\text{C}}^{X^{\prime }}{\text{W}}^{{\text{X}}^{\prime }}={\text{W}}^{{\text{X}}^{\prime }}\lambda^{{\text{X}}^{\prime }}. \end{array}$$

Coordinates **X′**(*t*) are transformed onto principal components and normalized by roots of eigenvalues (space-whitening the signal) to get flattened normalized projections:

(5) $$\text{Y}(t)={\left(\lambda^{X^{\prime }}\right)}^{-1/2}({\left({\text{W}}^{X^{\prime }}\right)}^{T}X^{\prime }(t)).$$

A time-lagged covariance matrix is calculated as:

(6) $$\begin{array}{r} C_{ij}^{\text{Y}}=\langle Y_{i}\left(t\right)Y_{j}\left(t+\tau \right)\rangle , \end{array}$$

where τ is an adjustable time lag. Because the matrix *C* is non-symmetric it must be symmetrized as:

(7) $$\begin{array}{r} {\text{C}}_{sym}^{\text{Y}}=1/2\left({\text{C}}^{\text{Y}}+{\left({\text{C}}^{\text{Y}}\right)}^{T}\right). \end{array}$$

Next, this symmetric matrix is decomposed to eigenvalues **λ**^**Y**^ and eigenvectors **W**^**Y**^:

(8) $$\begin{array}{r} {\text{C}}_{sym}^{\text{Y}}{\text{W}}^{\text{Y}}={\text{W}}^{\text{Y}}\lambda^{\text{Y}}. \end{array}$$

Finally, **Y**(*t*) are transformed onto principal components and expanded by eigenvalues:

(9) $$\begin{array}{r} \text{Z}={\left(\lambda^{\text{Y}}\right)}^{1/2}\left({\left({\text{W}}^{\text{Y}}\right)}^{T}\text{Y}\right). \end{array}$$

This step expands distances in directions with highest autocorrelations, which represent directions of rarely occurring transitions.

It is possible to use certain number of eigenvectors with highest eigenvalues instead of all eigenvectors. This selection may be driven by relaxation time decays (see Wehmeyer et al., 2019) but this is out of scope of this article.

t-SNE can be applied on distances between simulation snapshots calculated in the space of **Z** as:

(10) $$\begin{array}{r} D_{t,t^{\prime }}=||\text{Z}\left(t\right)-\text{Z}\left(t^{\prime }\right)||. \end{array}$$

Low-dimensional embeddings obtained in this step are further referred to as time-lagged t-SNE coordinates (t-t-SNE). For the sake of comparison, low-dimensional embedding obtained by standard TICA (without t-SNE step) and standard t-SNE (without TICA step) were also calculated and are further referred to as TICA coordinates and t-SNE coordinates, respectively. t-SNE and t-t-SNE coordinates are unit-free because they are set in order to fit the corresponding unit-free proximities (both *D* and σ in Equation 1 are measured in the same units). It must be kept in mind that t-SNE and t-t-SNE use random initiation of low-dimensional points, so recalculation leads to a different plot.

All analyses were done by programs written in Python with MDtraj (McGibbon et al., 2015) (for reading trajectories), PyEMMA (Wehmeyer et al., 2019) (for testing of algorithms), numpy (Oliphant, 2006) (to implement AMUSE algorithm) and scikit-learn (Pedregosa et al., 2011) (to run t-SNE) libraries. It is available at GitHub (https://github.com/spiwokv/tltsne) and using PyPI.

The trajectory of alanine dipeptide was obtained by unbiased 200 ns molecular dynamics simulation of a system containing alanine dipeptide and 874 TIP3P (Jorgensen et al., 1983) water molecules in Gromacs (Abraham et al., 2015). It was modeled by Amber99SB-ILDN force field (Lindorff-Larsen et al., 2010). Simulation step was set to 2 fs and all bonds were constrained by LINCS algorithm (Hess et al., 1997). Electrostatic interactions were treated by particle-mesh Ewald method (Darden et al., 1998). Temperature was kept constant (NVT ensemble) at 300 K by V-rescale thermostat (Bussi et al., 2007).

The trajectory of Trp-cage folding and unfolding was kindly provided by DE Shaw Research.

## 3. Results

The method was tested on two molecular systems—on alanine dipeptide and Trp-cage. In order to test time-lagged t-SNE we compare time-lagged t-SNE with standard t-SNE and TICA.

### 3.1. Alanine Dipeptide

Time-lagged t-SNE was first applied on a trajectory of alanine dipeptide without water and hydrogen atoms. It is important to remove hydrogen atoms because rotamers of methyl groups by approx. 120 deg are mathematically distinguishable but chemically identical. The trajectory was sampled every 20 ps (10,001 snapshots). Time lag τ was set to 3 frames (60 ps). The value of perplexity was set to 3.0 and Euclidean space was used to calculate the distance matrix **D**.

The value of lag time was chosen based on TICA results. Similar calculations with lag time set to 1 to 12 steps show that lag time set to 1–7 works well on a simple system such as alanine dipeptide (see Supplementary Material). All eigenvectors **W**^**Y**^ were used in Equation (9).

**Figure 1:**
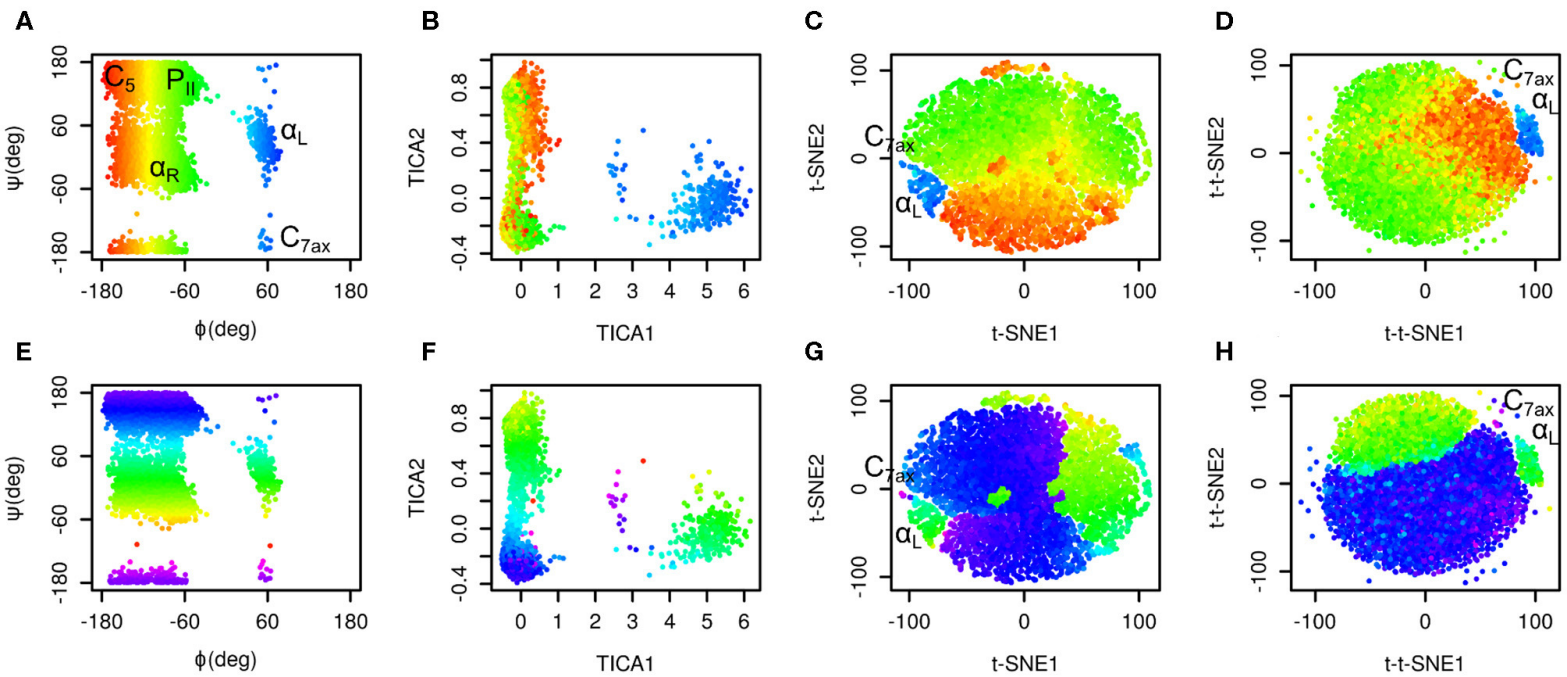
Time-lagged t-SNE (t-t-SNE) applied on 200 ns simulation of alanine dipeptide in water. Conformations sampled in the simulations were projected into the space of Ramachandran torsions ϕ and ψ **(A,E)**, TICA coordinates **(B,F)**, t-SNE **(C,G)** and time-lagged t-SNE **(D,H)**. Points are colored by Ramachandran torsion ϕ **(A–D)** and ψ **(E–H)**.

The results are depicted in Figure 1. Plots in the space of Ramachandran torsions show that all relevant conformations of alanine dipeptide were sampled. Plots in the space of TICA coordinates show that rotation around ϕ is the slowest and rotation around ψ is the second slowest motion in the studied system (slowest in terms of number of occurrences).

Plots in the space of t-SNE coordinates have a circular shape cut into multiple pieces by borders between different conformers. These plots show a limitation of conventional t-SNE, which is an improper resolution of conformations. Namely, there is a green island in the blue area of the plot colored by ϕ values (G).

Time-lagged t-SNE (t-t-SNE) does not suffer this problem. The blue area in the plot generated by time-lagged tSNE is continuous and does not contain any islands of conformations with positive ϕ values (H). This can be explained by the fact that introduction of a time lag into t-SNE causes higher separation of key conformations of alanine dipeptide.

One feature is common to the original t-SNE as well as our time-lagged variant. This is the fact that t-SNE flattens the distribution of points in the output space. This results in an almost uniform distribution of points in each minimum.

It is possible to calculate a histogram of some molecular collective variable or collective variables and convert it into a free energy surface. Most common interpretation of such free energy surfaces is that deep minima correspond to stable states, whereas shallow minima correspond to unstable states. This approach can be applied for conventional descriptors, such as Ramachandran angles of alanine dipeptide. However, due to flattening of distribution of points by t-SNE or by time-lagged t-SNE such free energy surface is relatively flat. Populations of different states can be estimated from areas of free energy minima rather than from their depths. In general, time-lagged t-SNE (as well as t-SNE) must be used with caution when applied to identify metastable states and to calculate free energy surfaces.

### 3.2. Trp-Cage

t-SNE and time-lagged t-SNE analysis was performed on the trajectory of Trp-cage folding and unfolding sampled every 20 ns (10,440 snapshots). Lag time was set to three frames (60 ns). Similarly to alanine dipeptide, lag time was chosen based on TICA analysis. Comparison of embeddings calculated for lag time set to 1, 2, 3, 4, 5, 10, 15, and 20 (in number of frames) shows that lag time 1–5 works well (see Supplementary Material).

Perplexity was set to 10.0. Several values were tested and perplexity set to 10 performs well in terms of the focus on local vs. global structure of data. Supplementary Material contains the results obtained for perplexity 5, 10, 20, 50, and 100. These results indicate that time-lagged t-SNE is relatively robust in terms of choice of perplexity and perplexity 10 and higher perform well.

Initial analysis by time-lagged t-SNE resulted in a circular plot with multiple points located outside clusters on the edges of the circle. This indicates that there are many points with high distances $D_{t,t^{\prime }}$. In order to eliminate these points we reduced the number of eigenvectors **W**^**Y**^ to top 50 eigenvectors (option -maxpcs in the code).

**Figure 2:**
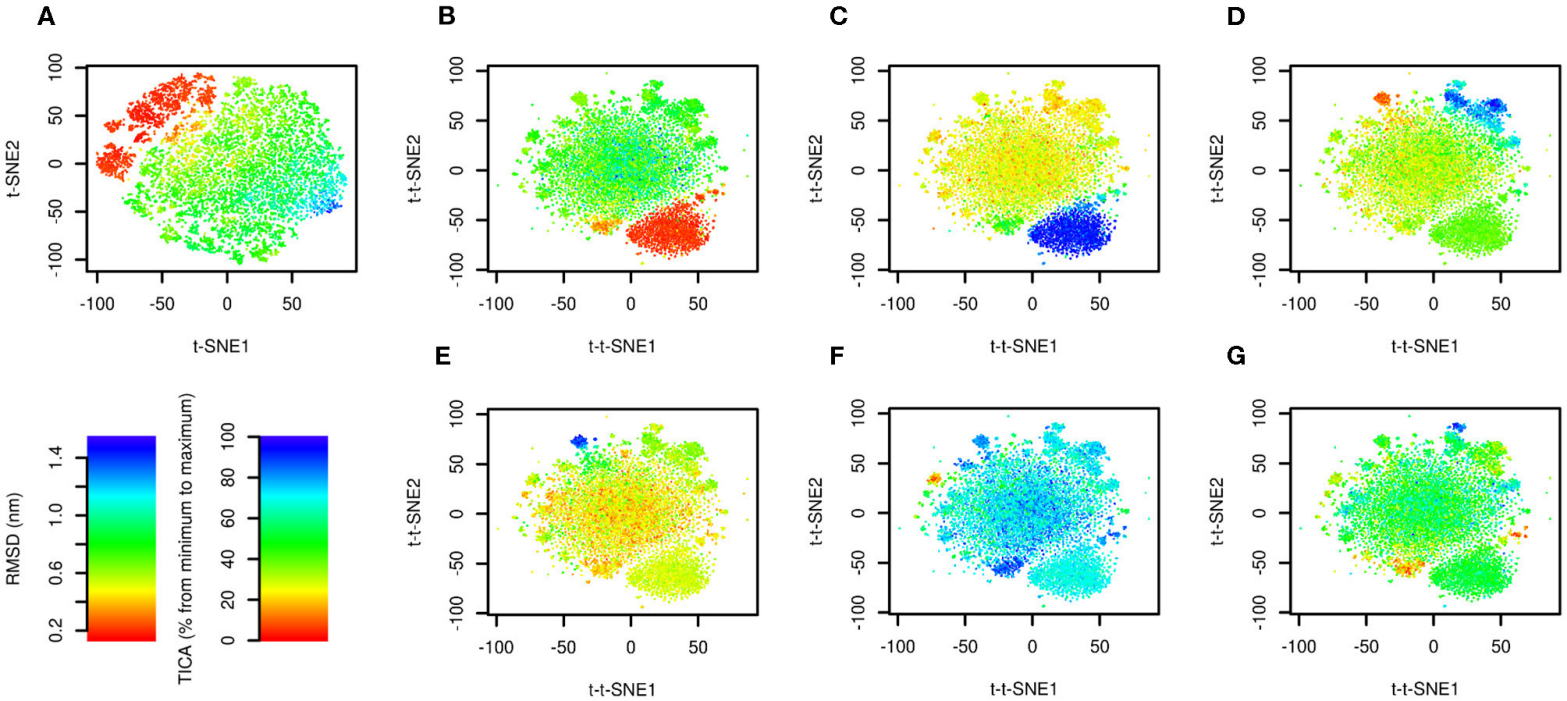
Time-lagged t-SNE (t-t-SNE) applied on 208.8 μs of Trp-cage folding and unfolding. The trajectory was analyzed by t-SNE **(A)** and time-lagged t-SNE **(B–G)**. Points are colored by RMSD from the native structure **(A,B)** and by the first **(C)**, second **(D)**, third **(E)**, fourth **(F)**, and fifth **(G)** TICA coordinate.

The results are depicted in Figure 2. Figure 2A shows the trajectory analyzed by conventional t-SNE colored by RMSD from the native structure (PDB ID: 1l2y, Neidigh et al., 2002). There is a clear relationship between t-SNE coordinates, in particular t-SNE1, and RMSD. The native structure (in red) forms a cluster in the top left corner of the plot. Structures with high RMSD (in blue) are characterized by highest values of t-SNE1.

The trajectory analyzed by time-lagged t-SNE colored by RMSD is depicted in Figure 2B. Similarly to Figure 2A the native structure forms a distinct cluster. In contrast to the conventional t-SNE, structures with high values of RMSD are scattered in the large cluster in the center. This indicates that transitions between high-RMSD structures are fast.

Figures 2C–G show the same plots colored by TICA coordinates. The first TICA coordinate (Figure 2C) distinguishes folded and unfolded structures. Plots colored by other TICA coordinates (Figures 2D–G) in most cases show a red or blue clusters on edges of the plot. This shows that time-lagged t-SNE captures rarely occurring transitions characterized by TICA, but more efficiently than TICA itself, because these motions can be depicted in a single plot.

**Figure 3:**
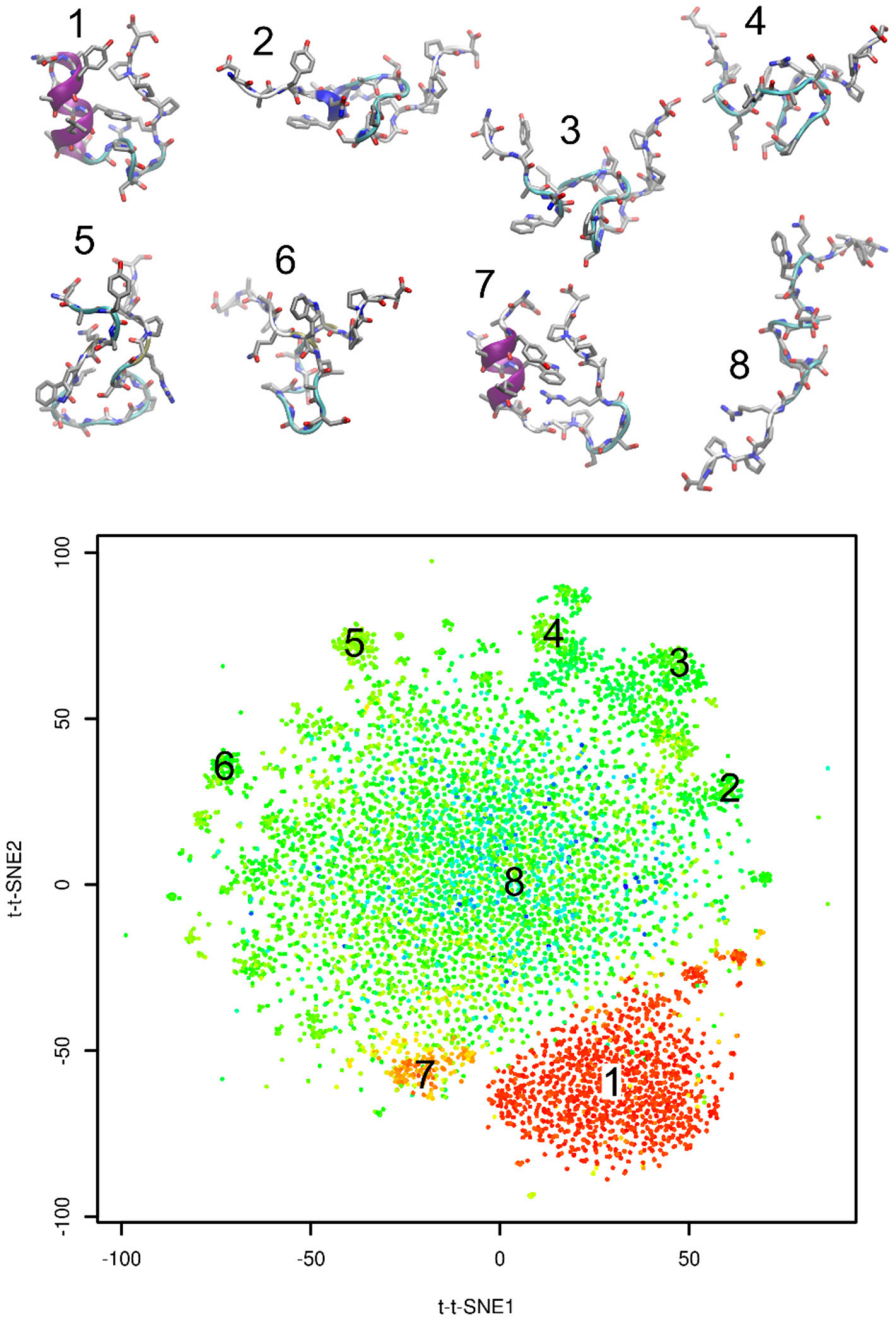
Representative structures projected onto time-lagged t-SNE embeddings. Plot is colored by RMSD from the native structure (as in Figure 2B).

Figure 3 shows representative structures of Trp-cage from the simulation trajectory projected onto time-lagged t-SNE embeddings. Structure 1 is the native structure. Structure 7 is a known near-native structure. Structures 2–6 were sampled from clusters on peripheral areas of time-lagged t-SNE embeddings. Finally, structure 8 was taken from the origin of the plot. Visual inspection indicates that structures 2–6 may be kinetic traps of Trp-cage folding, because these structures are characterized by formation of numerous non-native hydrogen bonds and other interactions. Also the near-native structure 7 is likely to be a kinetic trap of Trp-cage folding.

**Figure 4:**
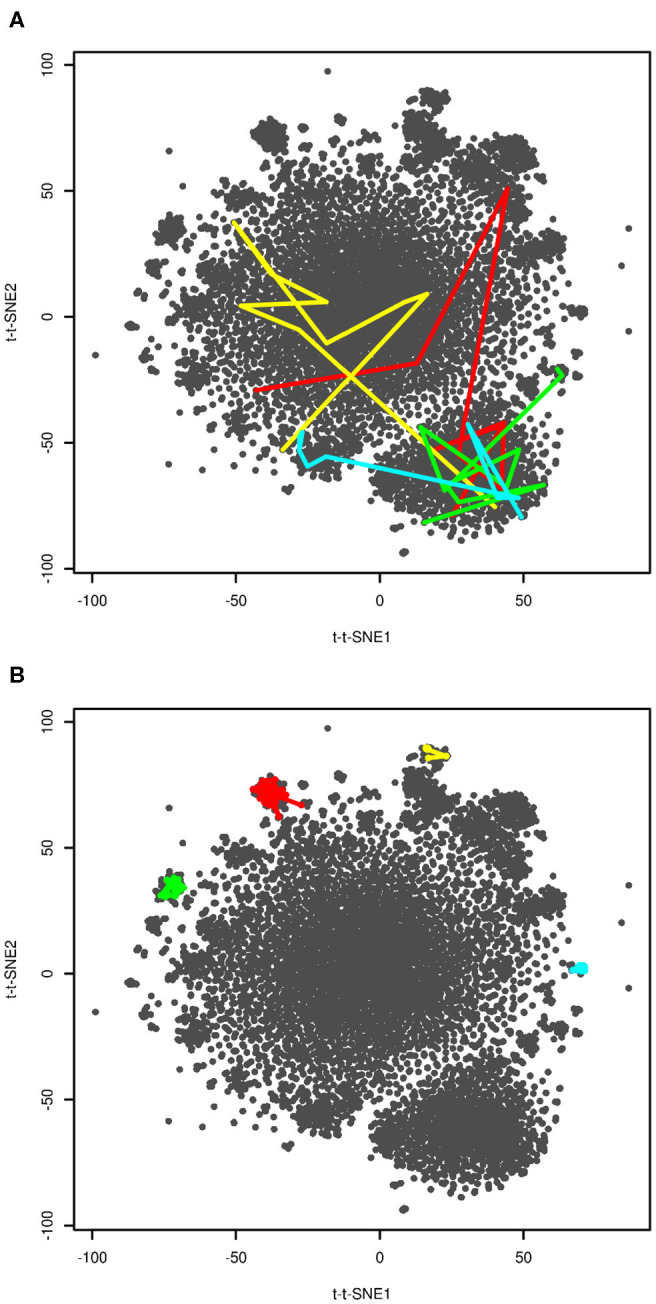
Visualization of folding events **(A)** and kinetic traps **(B)**. Four selected folding events (at approx. 20, 40, 104 and 206 μs in red, yellow, green, and blue, respectively) are depicted as last 10 frames (200 ns) before reaching t-t-SNE2 < −75. Four selected kinetic traps on edges of the plot are depicted as connected series of snapshots. These regions were sampled for 800 (red), 140 (yellow), 400 (green), and 460 (blue) ns.

In order to further interpret the plot we visualized four selected folding events. They are depicted in Figure 4A. These plots show snapshots sampled last 200 ns (10 snapshots) before folding. Unfortunately, we were not able to provide higher resolution of time, because this would require either analysis of a higher number of snapshots or recalculation of time-lagged t-SNE. The former was not possible due to computational costs, the latter due to impossibility of calculation of time-lagged t-SNE on out-of-sample structures (discussed later). Despite limited resolution of time, the plot shows that unfolded and folded structures are clearly separated. The fact that some folding processes passed clusters on edges of the plot close to the native structure may indicate that these clusters are near-native metastable states.

In previous paragraphs we interpreted clusters on edges of the plot (structures 2–6 in Figure 3). We investigated how long the system stayed in these regions. The results are shown in Figure 4B. The system stayed in these regions for 140–800 ns. This supports our interpretation of these regions as kinetic traps. Interestingly, all four regions depicted in Figure 4B were sampled multiple times in the simulation.

## 4. Discussion

Dimensionality reduction methods are frequently used to analyze data from biomolecular simulations. Linear methods such as PCA have been used for decades, whereas application of non-linear methods is relatively new. Various linear and non-linear dimensionality reduction methods have various advantages and disadvantages.

PCA and other linear methods are easy to use (no additional parameters have to be set), they realistically map densities of states from the high-dimensional to low-dimensional space and it is straightforward to calculate low-dimensional embedding for a new out-of-sample structure. On the other hand, their performance in visualization is low because they usually require three or more dimensions to separate key states of the studied system.

Non-linear methods perform much better in dimensionality reduction but mapping of densities may be distorted (this is the case of t-SNE and its time-lagged variant, which tend to flatten the output densities) and calculation of low-dimensional embeddings for a new out-of-sample structure is complicated. t-SNE is useful specially for visualization purposes.

Comparison of t-SNE and time-lagged t-SNE shows a great advantage of our variant. Figure 2A shows that t-SNE coordinates correlate with RMSD from the native structure. The yellow-green-blue cloud of non-native structures in this plot represents a pool of non-native conformations in which short-living and long-living states overlap. On the other hand, in the time-lagged t-SNE there are short-living states in the center and long-living states, including the native state, are located on the edges of the plot. In a single plot it is possible to distinguish multiple key long-living states.

There is a disadvantage of time-lagged methods in their dependence on the choice of lag time. Choice of lag time for time-lagged t-SNE was driven by TICA analysis. Values of 3 frames (60 ps, 0.03% of the whole trajectory) for alanine dipeptide and 3 frames (60 ns, 0.029% of the whole trajectory) for Trp-cage led to visually plausible low dimensional embeddings. This indicates that 0.03% of trajectory size is a good initial choice of lag time.

Another disadvantage of time-lagged t-SNE is in distortion of densities and impossibility to easily calculate low-dimensional embeddings for a new out-of-sample structure. As an alternative to time-lagged t-SNE it is possible to use time-lagged autoencoders recently reported by Wehmeyer and Noé (2018). Autoencoders are feed-forward neural networks with an hourglass-like architecture. The input signal (atomic coordinates or other features) from the input layer are transformed via hidden layers into the central bottleneck layer. Next, the signal from the bottleneck layer is transformed via hidden layers into the output layers. Parameters of the network are trained to obtain agreement between the input and output signal. The signal in the bottleneck layer represents a non-linear low-dimensional representation of the input signal. Unlike classical autoencoders, time-lagged autoencoders focus on the most rarely occurring transitions, not on the most intensive motions (Wehmeyer and Noé, 2018).

The clear advantage of autoencoders and their time-lagged variant is the possibility to calculate low-dimensional embeddings for a new out-of-sample structure. Extensive testing of time-lagged autoencoders in the original article (Wehmeyer and Noé, 2018) was possible owing to this fact. Time-lagged autoencoders can be trained on a training set and tested on a validation set, i.e., they can be evaluated by cross-validation. Furthermore, they can be trained on a small training set and then applied on a large set of input data. This is efficient since the training part is in general significantly more expensive than the calculation of embeddings on out-of-sample structures. Time-lagged autoencoders are useful for pre-processing of structural data for building of Markov state models.

There are limited options for calculation of t-SNE low-dimensional embeddings for out-of-sample structures. Therefore, t-SNE and time-lagged t-SNE are not suitable for pre-processing of the structural data. We see the advantage of time-lagged t-SNE (similarly to t-SNE) in visualization.

Time-lagged t-SNE in the current implementation also cannot be used as collective variables in simulations using bias force or bias potential because these methods require on-the-fly calculation of low-dimensional embeddings and their derivatives with respect to atomic coordinates. However, there are tools to approximate such low-dimensional embeddings (Spiwok and Králová, 2011; Sultan and Pande, 2018; Trapl et al., 2019).

One of key features of t-SNE is that it can reconstruct proximities and not distances in the low-dimensional output space. In time-lagged t-SNE this means that states separated by low energy barriers are close to each other. States separated by large energy barriers are far from each other, but time-lagged t-SNE does not attempt to preserve their distances accurately. This means that two close points in the time-lagged t-SNE plot can be connected by an energetically favorable path.

Another key feature of t-SNE is perplexity and the fact that t-SNE flattens the distribution of points in the output space. This is useful for visualization. For this reason t-SNE (as well as time-lagged t-SNE) must be used with caution as a pre-processing for calculation of free energy surfaces and for clustering. t-SNE can also create artificial clusters when perplexity is not set properly.

## Data Availability Statement

The datasets generated for this study can be found in the https://github.com/spiwokv/tltsne.

## Author Contributions

Both authors developed the method and wrote the manuscript. VS wrote codes and run simulations and analysis.

## Conflict of Interest

The authors declare that the research was conducted in the absence of any commercial or financial relationships that could be construed as a potential conflict of interest.
