# Supplementary material for: Time-Lagged t-Distributed Stochastic Neighbor Embedding (t-SNE) of Molecular Simulation Trajectories
Source: Front Mol Biosci. 2020 Jun 30;7:132. doi: 10.3389/fmolb.2020.00132 (PMC7344294; doi:10.3389/fmolb.2020.00132)
Supplement: Supplementary file 1 [file Data_Sheet_1.PDF]

Time-Lagged t-Distributed Stochastic Neighbor Embedding (t-SNE) of Molecular Simulation Trajectories

A Preprint

Vojtěch Spiwok
Department of Biochemistry and Microbiology
University of Chemistry and Technology, Prague
Technická 5, Prague 6, 166 28, Czech Republic
spiwokv@vscht.cz

Pavel Kříž
Department of Mathematics
University of Chemistry and Technology, Prague
Technická 5, Prague 6, 166 28, Czech Republic
krizp@vscht.cz

April 22, 2020

1 Supplementary Material

Supplementary figures Fig. S1, Fig. S2 and Fig. S3.

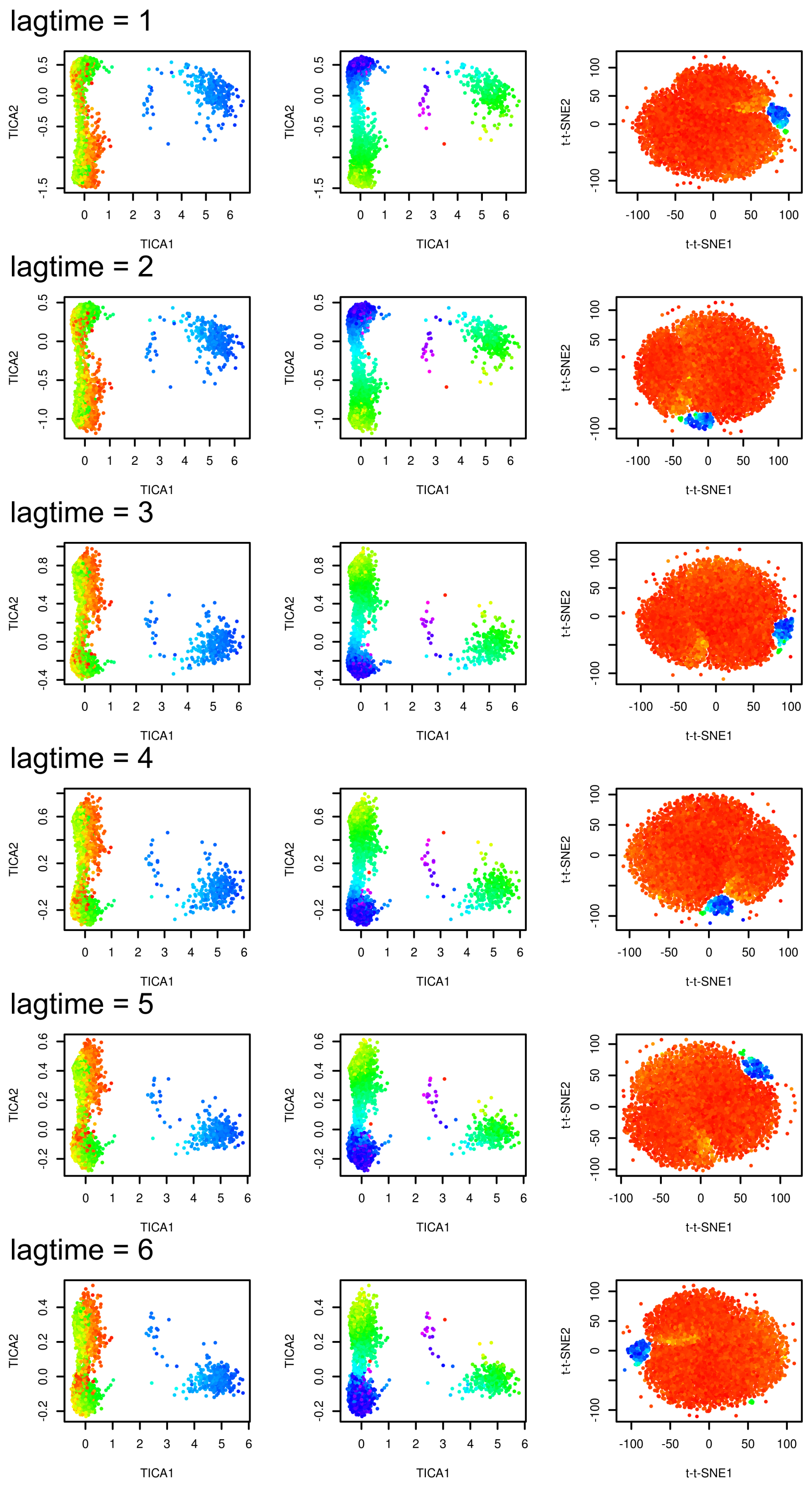

Figure S1: Effect of lag time on time-lagged t-SNE of alanine dipeptide trajectory. TICA plots are colored by ϕ (left) and ψ (centre, as in Fig. 1). Time-lagged t-SNE plots are colored by the first TICA coordinate (as in Fig. 1).

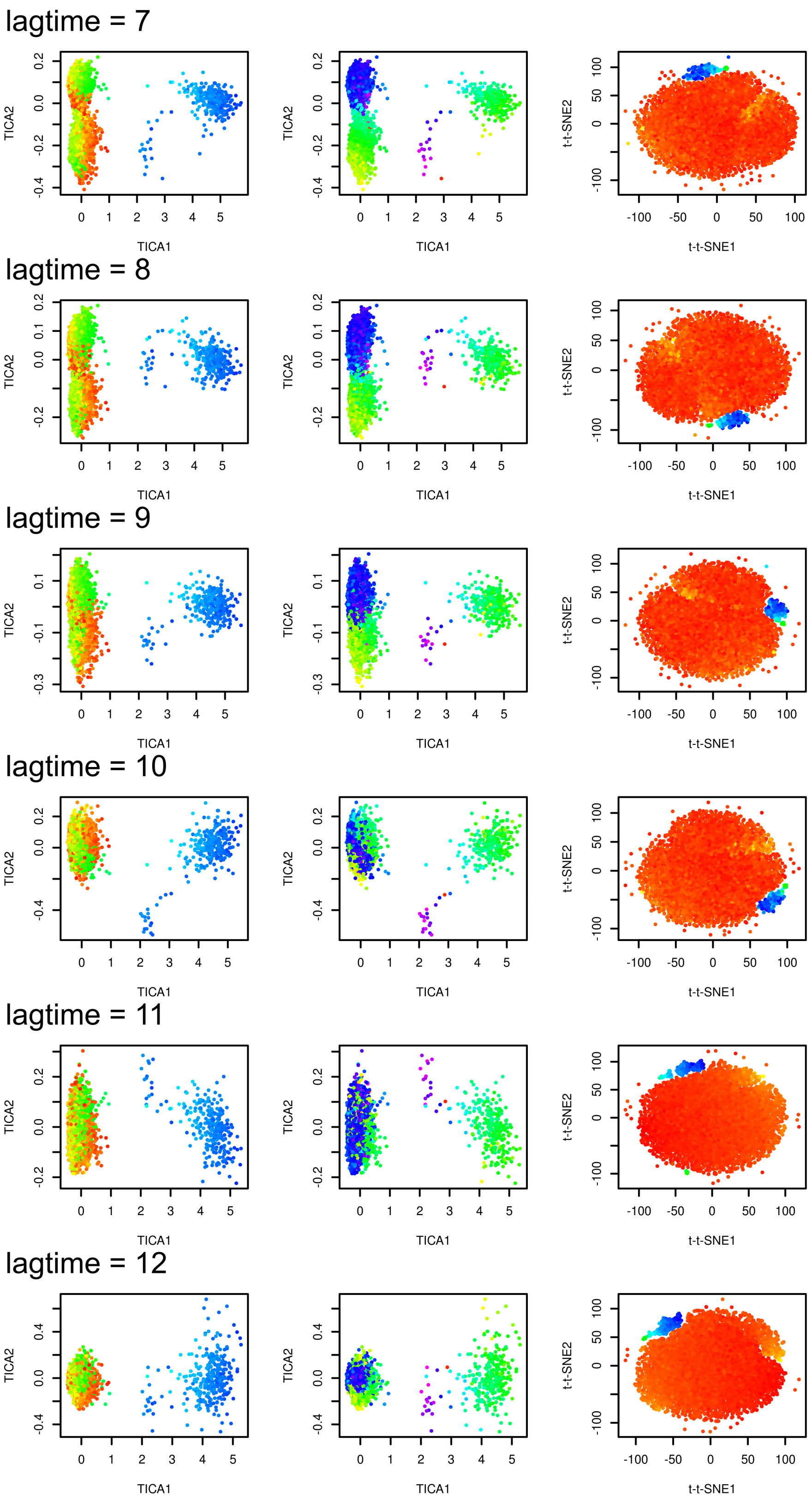

Figure S2: Effect of lag time on time-lagged t-SNE of alanine dipeptide trajectory. TICA plots are colored by ϕ (left) and ψ (centre, as in Fig. 1). Time-lagged t-SNE plots are colored by the first TICA coordinate (as in Fig. 1).

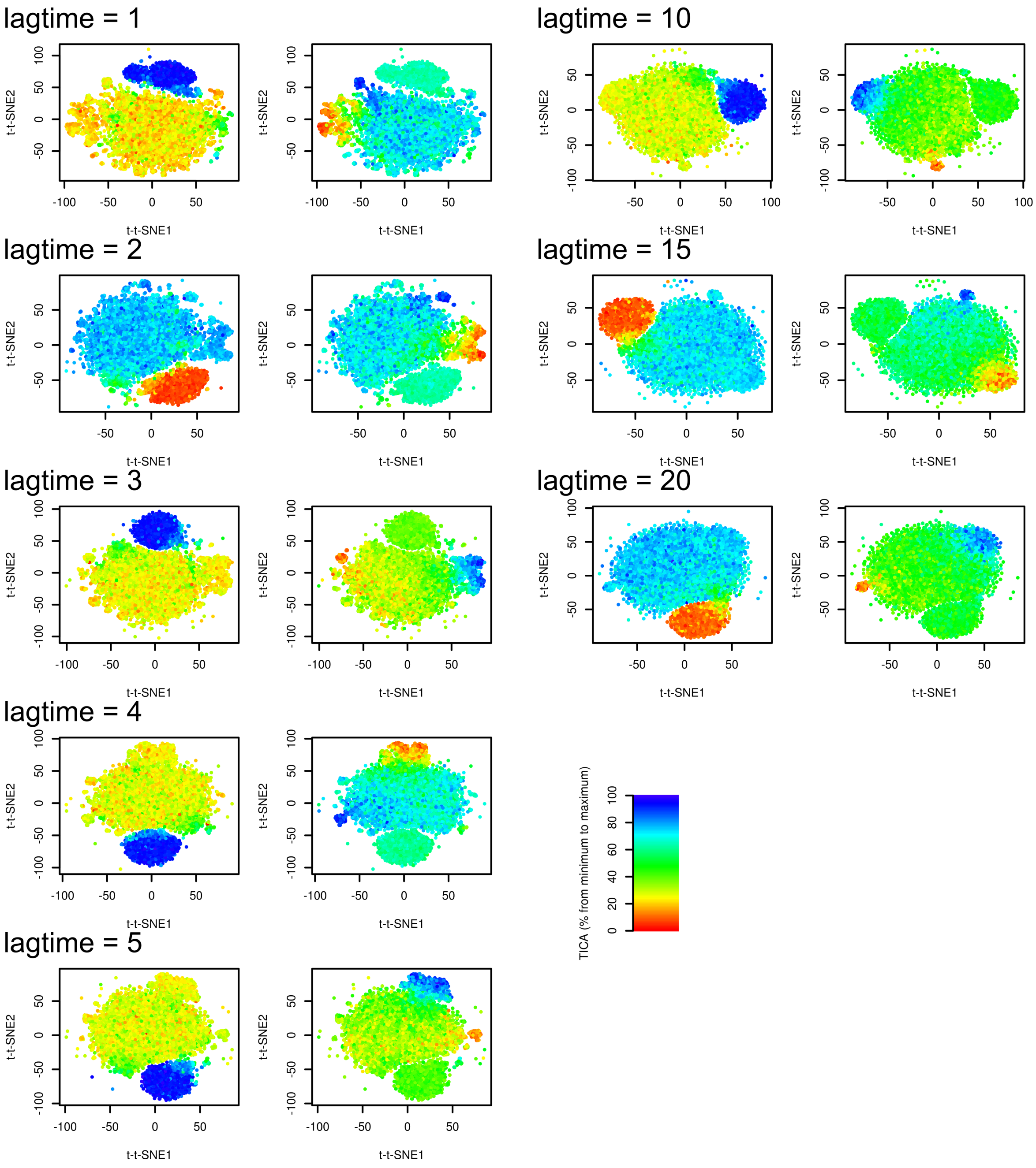

Figure S3: Effect of lag time on time-lagged t-SNE of Trp-cage folding trajectory. Plots are colored by the first and the second TICA coordinate (left and right, respectively).

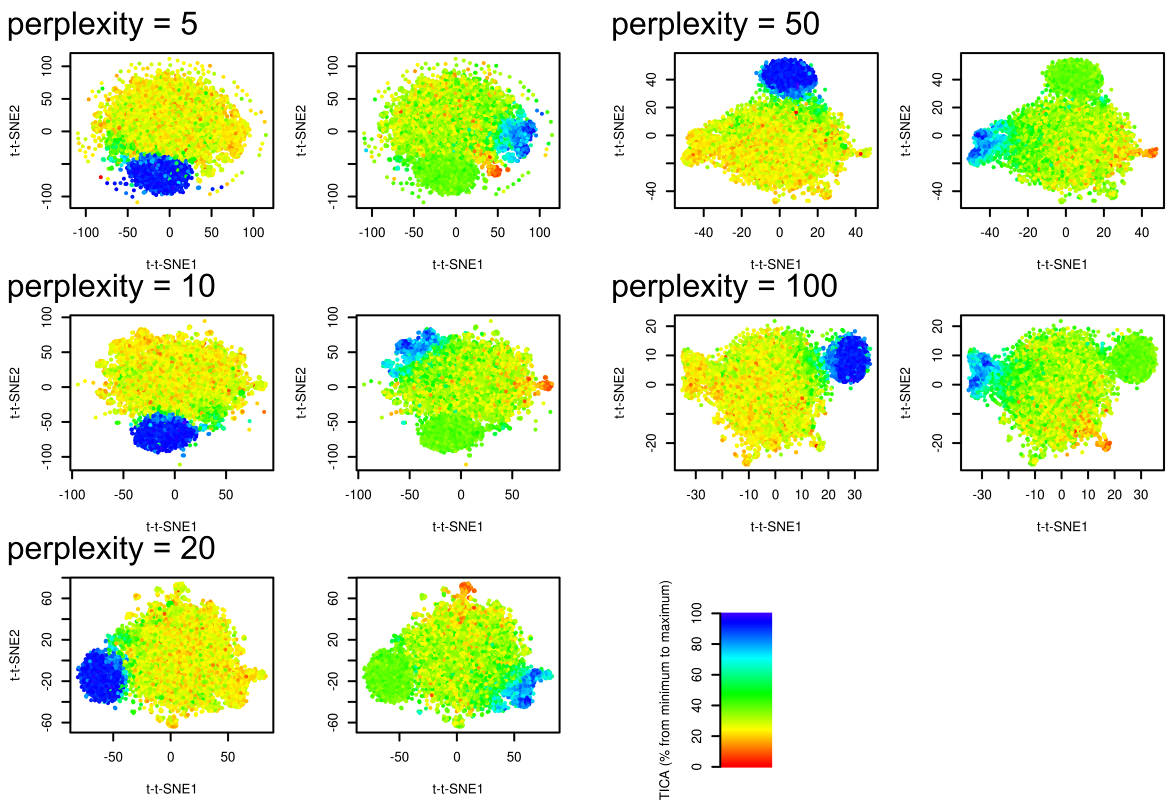

Figure S4: Effect of perplexity on time-lagged t-SNE of Trp-cage folding trajectory. Plots are colored by the first and the second TICA coordinate (left and right, respectively).
